# Associations of viral infections, including cutaneous warts, and sleep quality with immune function in treatment-naïve breast cancer patient: a cross-sectional study

**DOI:** 10.3389/fmed.2026.1886238

**Published:** 2026-09-15

**Authors:** Hong Xu, Lihua Xue, Hao Zheng, Yajing Wang, Xinwei Liu, Bu Da, Yanqi Ye, Shan Miao

**Affiliations:** 1 Senior Department of General Surgery, The 1st Medical Center, Chinese PLA General Hospital, Beijing, China; 2 Department of General Surgery, The Third Medical Center, Chinese PLA General Hospital, Beijing, China

**Keywords:** breast cancer, cutaneous warts, emotional state, immune function, lymphocyte count, sleep quality, viral infection

## Abstract

**Background:**

Viral infections and sleep disturbance may be associated with immune dysregulation, but their relationship with baseline immune status in treatment-naïve breast cancer remains unclear. We investigated associations of viral infection history, including cutaneous warts, and sleep quality with peripheral lymphocyte counts.

**Methods:**

This cross-sectional study included 100 women with pathologically confirmed breast cancer who had received no antitumor therapy. Sleep quality was assessed using the Pittsburgh Sleep Quality Index. Data on psychological status, systemic viral infections, cutaneous warts, and absolute lymphocyte counts were collected. Group comparisons and multivariable linear regression were performed.

**Results:**

Systemic viral infections were reported in 41% of patients, including herpes zoster (8%), cervical HPV infection (5%), Epstein–Barr virus infection (2%), respiratory syncytial virus infection (2%), hepatitis B virus carrier status (3%), and other/unspecified viral infections (21%). Cutaneous warts were present in 40, and 62% had at least one type of viral infection. Sleep disorders were present in 73%, while 23% had lymphocyte counts below 1.1 × 10^9^/L. Patients with cutaneous warts had lower lymphocyte counts than those without warts (1.58 ± 0.73 vs. 1.89 ± 0.86 × 10^9^/L, *p* < 0.05). Counts were also lower in patients with systemic viral infections than in those without (1.52 ± 0.71 vs. 1.92 ± 0.85 × 10^9^/L, *p* < 0.01), and were lowest in patients with both systemic infections and cutaneous warts (1.38 ± 0.64 × 10^9^/L, *p* < 0.001 vs. no infection). Patients with sleep disorders also had lower counts than those with acceptable sleep (1.58 ± 0.73 vs. 2.01 ± 0.88 × 10^9^/L, *p* < 0.01). Emotional distress was not independently associated with lymphocyte counts (*p* = 0.36). In multivariable analysis, sleep disorder (*β* = −0.18, *p* < 0.05) and viral infection history (*β* = −0.25, *p* < 0.01) were independently associated with lower lymphocyte counts.

**Conclusion:**

Viral infections, cutaneous warts, and sleep disturbance were common and associated with lower baseline lymphocyte counts in treatment-naïve breast cancer. However, because cutaneous warts and systemic viral infections were not entered as separate predictors, the independent contribution of cutaneous warts was not established. These findings should be considered exploratory and hypothesis-generating.

## 1 Introduction

Breast cancer is the most common malignancy among women in China, with approximately 357,200 new cases reported in 2022 (1). Its incidence continues to rise, and the age at onset appears to be shifting toward younger populations. The median age at diagnosis in China is 45–49 years, which is substantially younger than the 62–64 years reported in Western countries. Patients younger than 40 years account for 15–20% of all breast cancer cases in China, with an estimated annual increase in incidence of approximately 2% (2, 3). This epidemiological pattern suggests that studies of breast cancer etiology in Chinese populations should consider population-specific genetic backgrounds and environmental exposures.

Breast cancer arises from complex interactions among genetic susceptibility, hormonal factors, lifestyle-related factors, and environmental exposures; however, established risk factors do not fully explain a substantial proportion of cases. Recent advances in psychoneuroimmunology have increasingly highlighted the role of the neuroendocrine–immune network in tumorigenesis (4). Sleep disturbances and chronic psychological stress may impair immune function, thereby weakening antitumor immune surveillance (5). Notably, these factors may precede the diagnosis of breast cancer and contribute to a biological milieu that facilitates tumor development. Accordingly, integrated assessment of sleep quality, psychological status, and immune function may provide important insights into tumor biology and help inform the prediction of treatment responses.

The contribution of viral infections to carcinogenesis is well established. Established causal relationships exist between Epstein–Barr virus (EBV) and nasopharyngeal carcinoma/lymphoma, human papillomavirus (HPV) and cervical/oropharyngeal cancers, and hepatitis B/C viruses and hepatocellular carcinoma. In breast cancer, HPV, EBV, human cytomegalovirus (CMV), and mouse mammary tumor virus-like sequences have been detected in tumor tissues in an increasing number of studies, although the clinical significance of these findings remains controversial. Nevertheless, available evidence suggests potential associations between viral infections and certain breast cancer subtypes (6). Immune dysfunction may increase susceptibility to latent viral reactivation and new infections, whereas viral infections may promote persistent immune activation, T-cell dysfunction, and immune network dysregulation, potentially exacerbating immune impairment. These bidirectional interactions may contribute to the formation of a tumor-promoting microenvironment (7–9).

Cutaneous warts are caused by cutaneous HPV infection and represent one of the most common viral skin diseases. Their incidence is markedly increased in patients with primary immunodeficiency, organ transplant recipients receiving immunosuppressive therapy, and individuals with human immunodeficiency virus infection. The prevalence of cutaneous warts also increases with age (10–12). Therefore, cutaneous warts may serve as a readily identifiable clinical marker of cellular immune status. In patients with cancer, the presence of cutaneous warts may reflect pre-existing immune compromise. However, the prevalence of cutaneous warts in patients with breast cancer and their association with immune function have not been systematically investigated.

Based on this rationale, we conducted a cross-sectional study of 100 treatment-naïve patients with breast cancer to evaluate the associations among viral infections (including cutaneous warts), sleep quality, psychological status, and immune function as reflected by peripheral lymphocyte counts. Particular attention was given to the prevalence and potential clinical relevance of cutaneous warts. This study aimed to provide a clinical perspective for the comprehensive assessment of patients with breast cancer and to establish a basis for future studies investigating treatment response and prognosis.

## 2 Materials and methods

### 2.1 Study population

This study included 100 female patients with pathologically confirmed primary breast cancer who had not yet received any antitumor therapy (surgery, chemotherapy, radiotherapy, targeted therapy, or endocrine therapy). Patients were enrolled between 2024 and 2025 from the Breast Centers of the First and Third Medical Centers of the Chinese PLA General Hospital. Clinical data were collected after admission and prior to treatment initiation.

Inclusion criteria: (1) pathologically confirmed primary breast cancer; (2) no prior antitumor therapy; (3) complete clinical data; and (4) informed consent.

Exclusion criteria: (1) concurrent other malignancies; (2) known primary immunodeficiency; (3) receipt of immunosuppressive therapy within 3 months; and (4) acute infection phase.

Ethics statement: This study was approved by the Ethics Committee of the First Medical Center, Chinese PLA General Hospital. Written informed consent was obtained from all participants.

### 2.2 Data collection and definitions

#### 2.2.1 General clinical data

The data collected included age, body mass index (BMI), family history of breast cancer, reproductive history, breastfeeding history, age at menarche, age at menopause, and history of hormone use.

#### 2.2.2 Sleep quality assessment

Sleep quality over the past month was assessed using the Pittsburgh Sleep Quality Index (PSQI). A total PSQI score >5 was defined as sleep disorder. In addition to the PSQI, patients were asked to self-rate their sleep quality into five grades (“good,” “fair,” “poor,” “bad,” and “very bad”). For the dichotomous analysis, “good” and “fair” were considered acceptable sleep, while the other three grades were considered sleep disorders. This dual approach was used to capture both the standardized PSQI metric and a more granular, patient-centered assessment of perceived sleep quality. The five-grade self-report was used for the dose–response analysis (Table 1) to illustrate the graded relationship between perceived sleep quality and lymphocyte counts, while the PSQI-derived dichotomous variable was used in the multivariable regression model to maintain consistency with the established PSQI cutoff.

**Table 1:** Association between sleep quality and lymphocyte counts.

Analysis	Group	*n*	Lymphocyte count (10^9^/L)	*t*/*F*	*p*
Dichotomous	Sleep disorder group	73	1.58 ± 0.73	2.95	<0.01
	Acceptable sleep group	27	2.01 ± 0.88		
Five grades	Good	17	2.08 ± 0.91	8.9	<0.001^†^
	Fair	10	1.89 ± 0.82		
	Poor	28	1.68 ± 0.75		
	Bad	26	1.55 ± 0.71		
	Very bad	19	1.42 ± 0.66		

^†^*p* for trend.

#### 2.2.3 Psychological state assessment

Psychological state was recorded using self-assessments that included normal, mild anxiety, moderate anxiety, anxiety (ungraded), depression, and anger/irritability. These were dichotomized into “normal mood” and “emotional distress” (any anxiety, depression, or irritability) for analysis. The use of non-validated self-reports for psychological assessment is acknowledged as a limitation, as it may have introduced measurement error and contributed to the non-significant association observed in the multivariable analysis.

#### 2.2.4 Viral infection history

The systemic viral infection history consisted of documented non-cutaneous viral infections, including herpes zoster, cervical HPV infection, EBV infection, respiratory syncytial virus infection, and hepatitis B virus carrier status, and other/unspecified viral infections reported by patients. Information was obtained from patient self-reports and medical records. The “other/unspecified” category includes self-reported viral infections for which specific documentation was not available in the medical records, including but not limited to upper respiratory tract viral infections, gastrointestinal viral infections, and other common viral illnesses. The heterogeneity of this category is acknowledged as a limitation, and the results involving the combined “viral infection” variable should be interpreted with caution.

Cutaneous wart infection history consisted of documented or visible cutaneous warts, including common warts (hands/feet), flat warts (face/dorsum of hands), plantar warts (soles), and multi-site or multiple warts. Information was obtained from patient self-reports and physical examinations.

Patients were categorized into four subgroups: no viral infection, cutaneous warts only, systemic viruses only, and systemic viruses plus cutaneous warts. For patients with both cervical HPV infection and cutaneous warts, they were classified in the “systemic viruses + cutaneous warts” group, as cervical HPV was considered a systemic (non-cutaneous) viral infection. The immunological implications of systemic (mucosal) HPV and cutaneous HPV infection may differ due to distinct viral genotypes, tissue tropisms, and immune evasion mechanisms; this distinction is discussed as a limitation.

#### 2.2.5 Immune function indicator

The core indicator of cellular immune function was the peripheral blood absolute lymphocyte count. The normal reference range is 1.1–3.2 × 10^9^/L. White blood cell count was also recorded as a reference.

#### 2.2.6 Tumor characteristics

Due to the retrospective nature of data collection, tumor staging (TNM), hormone receptor status (ER/PR), HER2 status, and molecular subtype data were not systematically available for all patients. This limitation is discussed in the Limitations section, and the lack of these data is acknowledged as a potential confounder that may influence peripheral lymphocyte counts independently of the exposures of interest.

### 2.3 Statistical analyses

Statistical analyses were performed using SPSS version 26.0 (IBM Corp., Armonk, NY, USA). Continuous variables were expressed as means ± standard deviations (SDs). Comparisons between two groups were performed using independent-sample *t* tests. Comparisons among multiple groups were performed using one-way analysis of variance (ANOVA), with post-hoc pairwise comparisons using the least significant difference (LSD) method. We acknowledge that the LSD test is a liberal post-hoc method that does not adequately control for Type I error when making multiple comparisons. More conservative approaches such as Tukey’s Honestly Significant Difference (HSD) or Bonferroni correction are recommended for future studies (13). Given the exploratory nature of this study, no adjustment for multiple testing was applied; readers are cautioned that some significant findings may reflect Type I error, particularly in subgroup comparisons with small sample sizes. Categorical variables were expressed as counts and percentages, and group comparisons were performed using the chi-square test or Fisher’s exact test as appropriate. Multivariable linear regression was used to identify independent factors associated with lymphocyte count. Variables were entered based on their univariate significance (*p* < 0.10) and clinical relevance. This approach, while common, may introduce selection bias and is acknowledged as a limitation. Regression assumptions were assessed: normality of residuals was examined using the Shapiro–Wilk test (*p* = 0.18) and visual inspection of Q-Q plots, and multicollinearity was assessed using variance inflation factors (VIF; all VIF < 2.0), indicating acceptable model fit. The *R*^2^ and adjusted *R*^2^ values for the overall model are reported to indicate the proportion of variance explained. A two-sided *p* < 0.05 was considered statistically significant. No formal sample size calculation was performed *a priori*; a post-hoc power analysis indicated that with *n* = 100 and *α* = 0.05, the study had 80% power to detect a medium effect size (Cohen’s *f*^2^ = 0.15) for the regression model. Subgroup analyses with small sample sizes (e.g., combined groups with *n* < 20) should be interpreted as hypothesis-generating only.

## 3 Results

### 3.1 Participant characteristics

The mean age of the 100 patients was 52.8 ± 13.1 years (range: 28–79 years). The age distribution was: <40 years, 14; 40–49 years, 26; 50–59 years, 34; ≥60 years, 26. The mean BMI was 24.5 ± 4.2, with 41% normal weight, 30% overweight, and 21% obese. A family history of breast cancer was present in 12 participants (12%). Ninety-six patients (96%) had given birth, and 91 (91%) had breastfed. The mean age at menarche was 13.8 ± 1.9 years. A total of 58 participants (58%) were postmenopausal, with a mean age at menopause of 49.3 ± 3.6 years. Four patients (4%) had a history of hormone use.

### 3.2 Sleep quality

The PSQI assessment showed that 73% of patients (73/100) had varying degrees of sleep disturbance (self-rated as “poor,” “bad,” or “very bad”), while only 27% reported “good” or “fair” sleep quality (Table 2).

**Table 2:** Sleep quality distribution of patients with breast cancer (*n* = 100).

Sleep quality	Number of cases	Percentage (%)
Good	17	17
Fair	10	10
Poor	28	28
Bad	26	26
Very bad	19	19

### 3.3 Viral infection histories

The proportion of patients with a history of systemic viral infections (excluding cutaneous warts) was 41% (41/100). These included herpes zoster in eight cases (8%), cervical HPV infection in five cases (5%), EBV in two cases (2%), respiratory syncytial virus in two cases (2%), and hepatitis B virus carrier status in three cases (3%). The proportion of patients with a history of cutaneous wart infections was 40% (40/100), including common warts in 22 cases (22%), flat warts in 10 cases (10%), plantar warts in five cases (5%), and multi-site or multiple warts in three cases (3%). An additional 21 patients (21%) reported other/unspecified viral infections, including but not limited to upper respiratory tract viral infections, gastrointestinal viral infections, and other common viral illnesses. The proportion of patients with a history of cutaneous wart infections was 40% (40/100), including common warts in 22 cases (22%), flat warts in 10 cases (10%), plantar warts in five cases (5%), and multi-site or multiple warts in three cases (3%). The total proportion of patients any type of viral infection (including cutaneous warts) was 62% (62/100), and 19 cases (19%) had both systemic viruses and cutaneous warts. The comprehensive distribution is shown in Table 3.

**Table 3:** Comprehensive distribution of the viral infection histories (*n* = 100).

Category	Type	*n*	%
Systemic viral infections	Herpes zoster	8	8
	Cervical HPV infection	5	5
	Epstein–Barr virus	2	2
	Respiratory syncytial virus	2	2
	Hepatitis B virus carrier	3	3
	Other/unspecified	21	21
Cutaneous warts	Common warts (hands/feet)	22	22
	Flat warts (face/dorsum of hands)	10	10
	Plantar warts (soles)	5	5
	Multi-site/multiple warts	3	3
Comprehensive status	No viral infection	38	38
	Cutaneous warts only	21	21
	Systemic viruses only	22	22
	Systemic viruses + cutaneous warts	19	19

Other/unspecified viral infections include self-reported viral infections without specific documentation, such as upper respiratory tract viral infections, gastrointestinal viral infections, and other common viral illnesses. The heterogeneity of this category is acknowledged as a limitation.

### 3.4 Lymphocyte counts

The mean lymphocyte count was 1.75 ± 0.82 × 10^9^/L (range: 0.43–4.17 × 10^9^/L). The proportion of patients with lymphocyte counts below the normal lower limit (<1.1 × 10^9^/L) was 23% (23/100), indicating low cellular immune function in this subset (Table 4). Seven patients (7%) had severe lymphopenia with lymphocyte counts <0.8 × 10^9^/L.

**Table 4:** Lymphocyte counts distribution in patients with breast cancer (*n* = 100).

Lymphocyte count	Number of cases	Percentage (%)
Below normal limit (<1.1 × 10^9^/L)	23	23
Normal range (1.1–3.2 × 10^9^/L)	73	73
Above normal upper limit (>3.2 × 10^9^/L)	4	4

### 3.5 Association between viral infections and lymphocyte counts

Patients with cutaneous warts had significantly lower lymphocyte counts than those without (1.58 ± 0.73 vs. 1.89 ± 0.86 × 10^9^/L, *p* < 0.05). Patients with systemic viral infections had significantly lower lymphocyte counts than those without (1.52 ± 0.71 vs. 1.92 ± 0.85 × 10^9^/L, *p* < 0.01). Lymphocyte counts showed a decreasing trend (*p* < 0.001 for trend) as the number of distinct viral infection types increased. Patients with both systemic viruses and cutaneous warts had the lowest lymphocyte counts (1.38 ± 0.64 × 10^9^/L). The detailed data are shown in Table 5. Post-hoc pairwise comparisons for the combined effect analysis revealed that the “systemic virus + cutaneous warts” group had significantly lower lymphocyte counts than the “no viruses” group (*p* < 0.001), the “cutaneous warts only” group (*p* < 0.05), and the “systemic virus only” group (p < 0.05).

**Table 5:** Association between viral infections and lymphocyte counts.

Analysis	Group	*n*	Lymphocyte count (10^9^/L)	*t*/*F*	*p*
Cutaneous warts	With wart history	40	1.58 ± 0.73	2.21	<0.05
	Without wart history	60	1.89 ± 0.86		
Systemic viruses	With systemic viruses history	41	1.52 ± 0.71	2.89	<0.01
	Without systemic viruses history	59	1.92 ± 0.85		
Number of viral types	0 Types	38	1.98 ± 0.88	15.6^*^	<0.001^†^
	1 Type	34	1.69 ± 0.75		
	≥2 Types	28	1.45 ± 0.67		
Combined effect	No viruses	38	1.98 ± 0.88	7.2	<0.001
	Cutaneous warts only	21	1.72 ± 0.76		
	Systemic virus only	22	1.64 ± 0.72		
	Systemic virus + cutaneous warts	19	1.38 ± 0.64		

^*^ANOVA *F*-value; ^†^*p* for trend.

Post-hoc pairwise comparisons for combined effect: “systemic virus + cutaneous warts” vs. “no viruses”: *p* < 0.001; vs. “cutaneous warts only”: *p* < 0.05; vs. “systemic virus only”: *p* < 0.05.

### 3.6 Association between sleep quality and lymphocyte counts

Patients with sleep disorders had significantly lower lymphocyte counts than those with acceptable sleep (1.58 ± 0.73 vs. 2.01 ± 0.88 × 10^9^/L, *p* < 0.01). As sleep quality declined from “good” to “very bad,” lymphocyte counts decreased progressively from 2.08 ± 0.91 to 1.42 ± 0.66 × 10^9^/L (*p* < 0.001 for trend). The detailed data are shown in Table 1.

### 3.7 Association between psychological state and lymphocyte counts

Of the patients, 55% experienced emotional distress, with anxiety being the predominant type (48%). The lymphocyte count in the emotional distress group was lower than that in the normal mood group, but the difference did not reach statistical significance (1.68 ± 0.79 vs. 1.84 ± 0.85 × 10^9^/L, *p* = 0.16). The detailed data are shown in Table 6.

**Table 6:** Psychological state distribution and its association with lymphocyte counts.

Group	Number of cases	Lymphocyte count (10^9^/L)	*t*-value	*p*-value
Emotional distress group	55	1.68 ± 0.79	1.42	0.16
Normal mood group	45	1.84 ± 0.85		

### 3.8 Combined effect of sleep quality and viral infection

To further explore the relationship among the three factors, patients were cross-categorized by sleep quality (sleep disorder vs. acceptable sleep) and viral infection status (no viruses vs. viruses). The “sleep disorder + viruses” group had the lowest lymphocyte count (1.45 ± 0.67). This was significantly lower than that of the “acceptable sleep + no viruses” group (2.15 ± 0.93, *p* < 0.001) and also significantly lower than that of the “sleep disorder + no viruses” group (1.81 ± 0.79, *p* < 0.01). The detailed data are shown in Table 7.

**Table 7:** The combined effect of sleep quality and viral infection on lymphocyte counts.

Group	Number of cases	Lymphocyte count (10^9^/L)	95% CI	*p*-value^*^
Acceptable sleep + no viruses	12	2.15 ± 0.93	1.56–2.74	Reference
Acceptable sleep + viruses	15	1.89 ± 0.82	1.44–2.34	0.31
Sleep disorder + no viruses	26	1.81 ± 0.79	1.49–2.13	0.18
Sleep disorder + viruses	47	1.45 ± 0.67	1.25–1.65	<0.001

^*^“Viruses” refers to any type of viral infection (including cutaneous warts). *p*-values are from independent-sample *t* tests comparing each group to the “acceptable sleep + no viruses” reference group.

### 3.9 Multivariable linear regression analysis

Lymphocyte count was used as the dependent variable, and age, BMI, sleep disorder, presence of viral infection, and emotional distress were included in a multivariable linear regression analysis. Of note, this model used a combined “presence of viral infection” variable that did not separate cutaneous warts from systemic viral infections as independent predictors. The overall model was statistically significant (*F* = 5.82, *p* < 0.001) and explained 25.3% of the variance in lymphocyte counts (*R*^2^ = 0.253, adjusted *R*^2^ = 0.213). The results showed that after adjusting for age, BMI, and emotional state, the presence of sleep disorder (*β* = −0.18, *p* < 0.05) and viral infection history (*β* = −0.25, *p* < 0.01) remained independent influencing factors of lymphocyte counts. Emotional distress was not significantly associated with lymphocyte counts in the adjusted model (*β* = −0.07, *p* = 0.36). The detailed data are shown in Table 8.

**Table 8:** Multiple linear regression analyses of factors that influence the lymphocyte counts.

Variable	*B*	SE	*β*	*t*-value	*p*-value
Constant	2.35	0.31	–	7.58	<0.001
Age	−0.008	0.005	−0.13	−1.52	0.13
BMI	−0.012	0.016	−0.06	−0.75	0.46
Sleep disorder	−0.31	0.14	−0.18	−2.21	<0.05
Presence of viral infection	−0.42	0.13	−0.25	−3.23	<0.01
Emotional distress	−0.12	0.13	−0.07	−0.92	0.36

Overall model: *F* = 5.82, *p* < 0.001; *R*^2^ = 0.253, Adjusted *R*^2^ = 0.213.

## 4 Discussion

In this study, we systematically evaluated the prevalence of viral infections (including cutaneous warts) in patients with treatment-naïve breast cancer. The results revealed that 62% of these patients had at least one type of viral infection, with cutaneous warts accounting for 40% and systemic viral infections for 41%; 19% had both. These rates are substantially higher than those reported in the general population. This result highlights the potential clinical relevance of viral infections in this population.

This is the first study to systematically assess the distribution of cutaneous warts in patients with breast cancer. The results showed a cutaneous wart prevalence of 40%, which is 3–5 times higher than the 7–12% reported in the general population (14). However, it should be noted that the general population prevalence estimates are predominantly derived from younger cohorts (e.g., college students), and the prevalence of cutaneous warts is known to increase with age. Therefore, the comparison between our breast cancer cohort and general population data should be interpreted with caution. Cutaneous warts result from cutaneous HPV infection, and their occurrence, persistence, and recurrence are closely linked to T-cell-mediated cellular immunity. Arakawa et al. (8) demonstrated that T-cell-mediated immune attack is involved in the regression of flat warts. Genetic immunodeficiency studies have provided further evidence. Horev et al. (9) reported that IL-7 deficiency leads to autosomal recessive combined immunodeficiency, characterized by early-onset generalized warts and T-cell lymphopenia. Arango-Franco et al. (10) confirmed that IL-7 signaling is critical for T-cell development (14). In our study, patients with cutaneous warts had significantly lower lymphocyte counts than those without (*p* < 0.05). This result supports that cutaneous warts may serve as a simple external indicator of cellular immune status. However, it is important to note that in the multivariable regression model that included a combined “presence of viral infection” variable, the independent contribution of cutaneous warts specifically, after controlling for systemic viral infections and other confounders, was not formally established. As a result, the conclusion that cutaneous warts independently predict lower lymphocyte counts requires confirmation in future studies that enter cutaneous warts and systemic viral infections as separate independent variables in regression models.

Herpes zoster was detected in 8% of patients in the present study, which is significantly higher than the annual incidence in the general population. Herpes zoster results from the reactivation of latent varicella-zoster virus, and its occurrence is a clinical marker of declining cellular immunity. Hansson et al. (13) found that breast cancer was associated with a 1.29-fold increased risk of herpes zoster (95% CI 1.27–1.32). CMV has a seroprevalence of 70–90% in the general population. Studies have indicated that CMV encodes cmvIL-10 with potent immunosuppressive activity, which may influence the local immune microenvironment in breast cancer (16).

We introduced the concept of the number of distinct viral infection types in patients with breast cancer and observed a clear dose–response relationship. Lymphocyte counts decreased progressively from 1.98 ± 0.88 × 10^9^/L in those with no viral infections to 1.69 ± 0.75 × 10^9^/L in patients with one type, and to 1.45 ± 0.67 × 10^9^/L in those with two or more types (*p* < 0.001 for trend). Patients with both systemic viruses and cutaneous warts had the lowest lymphocyte counts (1.38 ± 0.64). This result suggests that different viral infections may exert synergistic pressure on the immune system, potentially contributing to immune dysregulation. However, we acknowledge that the term “immune exhaustion” typically requires supporting evidence from additional immunological markers such as CD4/CD8 ratio, NK cell counts, PD-1, TIM3, or cytokine profiles, which were not assessed in this study.

The 73% prevalence of sleep disorders in our cohort aligns with results from the literature (15). We found a significant dose–response relationship between sleep quality and lymphocyte counts (*p* < 0.001 for trend). The mechanisms by which sleep affects immune function are multifaceted, including neuroendocrine regulation, sympathetic nervous system activation, cytokine network dysregulation, and circadian rhythm disruption (17–21).

Emotional distress was present in 55% of patients, with anxiety being the predominant type (48%). Psychological stress can affect immune function by activating the hypothalamic–pituitary–adrenal axis and the sympathetic nervous system, and by causing behavioral changes (22). Although we did not find a significant association between emotional state and lymphocyte counts, the high prevalence of emotional distress underscores the importance of psychological support. The null finding may be attributable to several factors: (1) the use of non-validated self-report measures for psychological assessment, which may have introduced measurement error; (2) potential collinearity between sleep disorder and emotional distress, as these two variables are known to be closely interrelated (indeed, in our cohort, 78% of patients with emotional distress also had sleep disorders); and (3) the relatively small sample size may have limited statistical power to detect a modest effect. Future studies using validated psychological instruments and larger cohorts are needed to more definitively examine this relationship.

We propose an exploratory model based on our findings and the literature. Sleep disorders may initiate immunosuppression; immunosuppression may permit viral reactivation or new infections; viral infections may further exacerbate immune dysregulation; and immune dysregulation may increase susceptibility to infections and potentially tumor progression. The observation that patients with both sleep disorders and viral infections had the lowest lymphocyte counts (1.45 ± 0.67) provides preliminary clinical evidence in support of this model.

Our findings may have several clinical implications. First, routine assessment of sleep quality may help identify patients with breast cancer who are at risk of immune dysregulation. Second, the presence of cutaneous warts should not be regarded merely as a benign dermatological condition, but may serve as a visible clue to impaired cellular immunity, although this finding requires confirmation in studies that specifically isolate the effect of cutaneous warts from other viral infections by including them as separate predictors in multivariable models. Third, documenting the number and types of viral infections may provide additional information for identifying patients with potentially higher immune vulnerability. Fourth, psychological support should be considered an important component of comprehensive care for patients with breast cancer, given the high prevalence of emotional distress observed in this cohort and the well-documented effects of psychological stress on immune function in the broader literature (23) suggest that psychological support should still be considered an important component of comprehensive care for patients with breast cancer. The authors emphasize that this recommendation is primarily informed by the existing literature rather than by the null findings of this study.

### 4.1 Limitations

This study has several limitations that should be carefully considered when interpreting the results. First, the single-center cross-sectional design with a modest sample size (*n* = 100) limits the generalizability of the findings to broader breast cancer populations and precludes any causal inferences. The relatively small sample size may also have reduced statistical power to detect smaller effect sizes, particularly for the association between emotional distress and lymphocyte counts. While post-hoc power analysis suggested adequate power for the primary regression model, subgroup analyses with small sample sizes (e.g., “systemic virus + cutaneous warts,” *n* = 19; “acceptable sleep + no viruses,” *n* = 12) should be interpreted as hypothesis-generating only. Second, the retrospective collection of viral infection histories, based on patient self-reports and medical records, is subject to recall bias and incomplete documentation. This may have led to an underestimation of true infection prevalence. Third, the “other/unspecified” viral infection category (21% of patients) represents a heterogeneous group of self-reported infections without documented confirmation, which limits the interpretability of the combined viral infection variable in regression analyses. Fourth, sleep quality and emotional state were assessed using self-reports, with psychological status evaluated using non-validated instruments, which may have introduced measurement error and influenced the results. Furthermore, sleep disorder and emotional distress are known to be closely interrelated, and potential collinearity between these two variables may have influenced the regression results; indeed, 78% of patients with emotional distress in our cohort also had sleep disorders. Fifth, immune function was assessed solely using total lymphocyte counts, without detailed immunophenotyping (e.g., CD4/CD8 ratio, NK cell counts) or functional assays, which limits the depth of immune characterization. Sixth, the lack of tumor staging and molecular subtype data prevents analysis of potential confounding or effect modification by these important clinical factors. Tumor burden (stage) and tumor biology (hormone receptor status, HER2 status, molecular subtype) may independently influence peripheral lymphocyte counts and should be controlled for in future studies. Seventh, no HPV typing was performed for cutaneous warts, which would have allowed for more precise characterization of the infections and differentiation from mucosal HPV types. Eighth, the absence of longitudinal follow-up means that the clinical implications of these findings for treatment response, recurrence, or survival remain unknown. Ninth, the use of the LSD method for post-hoc comparisons does not adequately control for Type I error; more conservative methods such as Tukey’s HSD or Bonferroni correction should be employed in future studies (13). Given the exploratory nature of this study, no correction for multiple testing was applied, and readers are cautioned that some significant findings may reflect Type I error. Tenth, the approach of entering variables into the regression model based on univariate significance (*p* < 0.10) and clinical relevance, while common, may have introduced selection bias. Eleventh, and most importantly, the multivariable regression model included a combined “presence of viral infection” variable rather than separating cutaneous warts from systemic viral infections. This analytical choice limits the ability to draw conclusions about the independent contribution of cutaneous warts to lymphocyte counts after controlling for systemic viral infections. Consequently, one of the central conclusions of this study—that cutaneous warts may serve as an independent clinical indicator of impaired cellular immunity—requires confirmation in future studies that employ separate predictor variables. Twelfth, cervical HPV infection was categorized as a systemic viral infection, while cutaneous warts (also HPV-mediated) were treated as a separate category. These infections involve different viral genotypes, anatomical sites, and immune responses, and this distinction may have implications for interpreting the immunological findings. Future prospective, multicenter cohort studies with larger sample sizes, validated psychological instruments, comprehensive immune profiling, tumor characteristic data, HPV typing, and separate analysis of different viral infection types are needed to validate these associations and explore their implications for treatment responses and long-term outcomes.

## 5 Conclusion

In patients with treatment-naïve breast cancer, viral infections (including cutaneous warts) and sleep disorders are common and correlate with baseline immune function indicators. Cutaneous warts were found to be present in 40% of patients and may serve as a simple external indicator of a patient’s cellular immune status. Although the independent contribution of cutaneous warts requires further validation in regression models that separate them from systemic viral infections. Sleep disorders and viral infections may have synergistic negative effects on immune function. These results provide baseline clinical evidence for a potential association between cumulative viral burden and immune dysregulation, and suggest that comprehensive assessments of viral infection histories, sleep quality, and emotional state could contribute to more holistic evaluations of patients with breast cancer. The role of emotional distress, while not independently associated with lymphocyte counts in this study, remains clinically relevant based on its high prevalence and its well-established effects in the broader literature. Prospective cohort studies are still required to validate these associations and explore their implications for treatment responses and long-term outcomes.

## Data Availability

The original contributions presented in the study are included in the article/supplementary material, further inquiries can be directed to the corresponding author.
